# Genome Editing in Medicine: Tools and Challenges

**DOI:** 10.15388/Amed.2021.28.2.8

**Published:** 2021-08-17

**Authors:** Gunda Petraitytė, Eglė Preikšaitienė, Violeta Mikštienė

**Affiliations:** Department of Human and Medical Genetics, Institute of Biomedical Sciences, Faculty of Medicine, Vilnius University, Vilnius, Lithuania; Department of Human and Medical Genetics, Institute of Biomedical Sciences, Faculty of Medicine, Vilnius University, Vilnius, Lithuania; Department of Human and Medical Genetics, Institute of Biomedical Sciences, Faculty of Medicine, Vilnius University, Vilnius, Lithuania

**Keywords:** biological therapy, genome editing, DNA changes, gene therapy

## Abstract

Studies which seek fundamental, thorough knowledge of biological processes, and continuous advancement in natural sciences and biotechnology enable the establishment of molecular strategies and tools to treat disorders caused by genetic mutations. Over the years biological therapy evolved from using stem cells and viral vectors to RNA therapy and testing different genome editing tools as promising gene therapy agents. These genome editing technologies (Zinc finger nucleases, TAL effector nucleases), specifically CRISPR-Cas system, revolutionized the field of genetic engineering and is widely applied to create cell and animal models for various hereditary, infectious human diseases and cancer, to analyze and understand the molecular and cellular base of pathogenesis, to find potential drug/treatment targets, to eliminate pathogenic DNA changes in various medical conditions and to create future “precise medication”. Although different concerning factors, such as precise system delivery to the target cells, efficacy and accuracy of editing process, different approaches of making the DNA changes as well as worrying bioethical issues remain, the importance of genome editing technologies in medicine is undeniable. The future of innovative genome editing approach and strategies to treat diseases is complicated but interesting and exciting at once for all related parties – researchers, clinicians, and patients.

## Abstract (lt)

**Genomo redagavimas medicinoje: įrankiai ir iššūkiai:**

**Santrauka. **Tyrimai, kuriais siekiama įgyti fundamentinių žinių ir nuodugniau suprasti biologinius procesus, bei nuolatinė gamtos mokslų ir biotechnologijų pažanga teikia galimybių tyrėjams gydyti genetinių pokyčių nulemtus sveikatos sutrikimus pasitelkus molekulinius įrankius. Biologinė terapija per pastaruosius dešimtmečius nuolat vystėsi: nuo kamieninių ląstelių ir virusinių vektorių naudojimo iki RNR terapijos bei galiausiai iki genų terapijos, kurioje taikomos genomo redagavimo technologijos. Šios genomo redagavimo technologijos (cinko pirštų nukleazės, TAL efektorių nukleazės), ypač CRISPR-Cas sistema, lėmė perversmą genų inžinerijos srityje. Pastaroji sistema šiuo metu taikoma kuriant įvairių paveldimų, infekcinių ir vėžinių ligų ląstelių ir gyvūnų modelius; analizuojant ir siekiant suprasti molekulinius ir ląstelinius patogenezės procesus ir kartu ieškant gydymui ir vaistams potencialių taikinių; taisant patogeninius DNR sekos pokyčius ir kuriant ateities „tiksliuosius vaistus“. Nors veiksniai, kaip antai: tikslus redagavimo sistemos pristatymas į norimas ląsteles, redagavimo proceso veiksmingumas, skirtingi DNR pokyčio įtraukimo būdai, taip pat nerimą keliančios bioetinės problemos, trikdantys sklandų genomo terapijos taikymą, išlieka, genetinės redagavimo technologijos yra neginčijamai svarbios medicinoje. Inovatyvių genomo redagavimo metodų ir strategijų gydant ligas laukia sudėtinga, tačiau įdomi ateitis, svarbi visiems su sveikatos priežiūra susijusiesiems – tyrėjams, gydytojams ir pacientams.

**Raktažodžiai: **biologinė terapija, genomo redagavimas, DNR pokyčiai, genų terapija.

## Introduction

Evolution has provided many advantages beneficial to humankind in terms of achieving capabilities allowing to be superior over other species. The forces of natural selection acted mainly through the genomes of organisms introducing genetic changes that allowed to gain or lose certain functions. Unfortunately, not all the mutations are advantageous – many of them cause particularly serious, devastating, and life-threatening conditions. Currently from 6000 to 8000 rare hereditary disorders are defined ([1], also visit Orphanet database). Moreover, it is estimated that approximately 265 novel rare hereditary disorders are described every year [1] pointing to many undiscovered hereditary conditions waiting to be named in the future. For the most part of genetic disorders, effective and early diagnostics, treatment, and appropriate surveillance are demanded to maintain valuable human life.

To extensively understand and precisely treat disorders caused by genome mutations, molecular strategies and tools are necessary. This emphasizes the importance of rapid advances in various fields of science and technology. Interaction between different disciplines (namely, natural sciences, engineering, and technology) created perfect conditions to emerge genetic engineering in biotechnology which plays a significant role in medicine, too. Using genome editing, a revolutionizing genetic engineering technique for the DNA manipulation, different model organisms are being modified and animal models are created to explain the pathogenesis of various human diseases. To mention a few, severe combined immunodeficiency (SCID) is one of the immune system conditions modeled in marmosets [2], a neuromuscular disorder, called Duchenne muscular dystrophy, modeled in rats using genome editing [3], as well as miniature pig model for Laron syndrome [4] and many others. The accuracy of knowledge about pathology causing mechanism, which at least partly can be resolved by applying genome editing tools for animal disease modeling, determines the ability to understand its manifestation and to create proper medication. The treatment (medication), in the light of biotechnology, includes not only pharmacological substances but also biological therapy.

The clinical application of the genome editing tools in biological therapy emerged as a natural wish to correct (treat) the genetic mistakes causing specific phenotypes. Over the last few decades, the interest in the DNA correction by molecular editing led to an increasing number of experimental studies designed to master genome editing. Although intensive work built a solid knowledge about mechanism of several major genome editing tools, the more challenging and less predictable part of research is manipulating the genome of live human cells where precise correction is preferred. To determine the possibilities of genome editing technologies in treating diseases and further developing “genome editing medication”, the understanding of existing biological therapy including genome editing tools, different approaches of making the DNA change as well as challenges of using genome editing in humans is required. 

## The development of biotherapy enables progress in genome editing

### Stem cell therapy and antisense oligonucleotides

**Figure 1.**
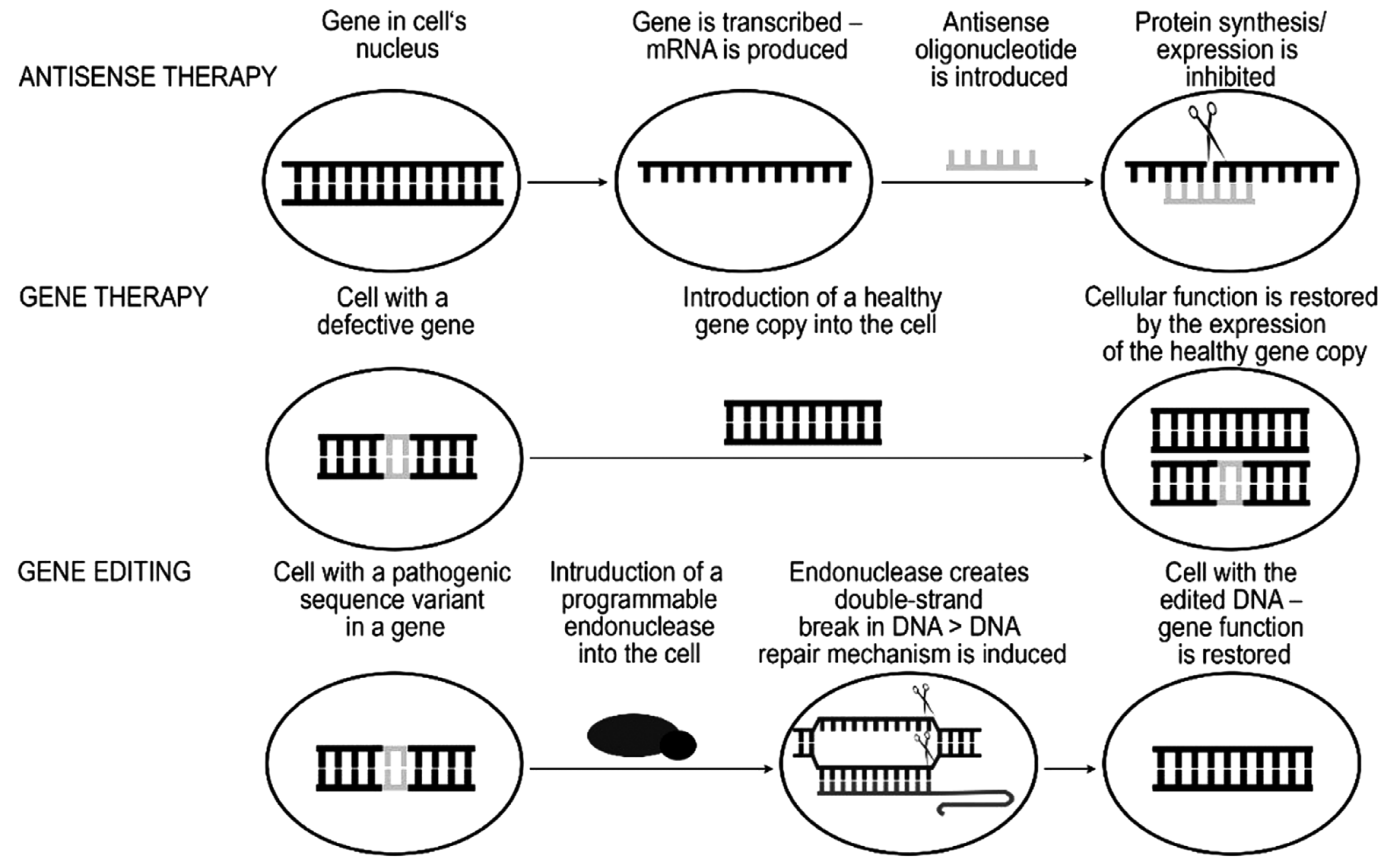
**The principle of antisense technology, gene therapy and gene editing. **In the antisense therapy RNA oligonucleotides (antisense oligonucleotides) are used to inhibit or decrease the protein synthesis by targeting the mRNA of the gene encoding the protein. Gene therapy is based on introducing an additional copy of a healthy gene to restore the cell function. Gene editing technology allows to directly target the DNA sequence of interest and to correct the genomic sequence variant.

**Stem cell therapy** (bone marrow transplantation in the late 1960s and early 1970s) was the first step in the concept of treatment where damaged, pathological cells (or biomolecules) are replaced with healthy ones [5]. The main difficulty in this type of therapy is finding an HLA-matched donor for transplantation and the subsequent risk of organ/cells rejection. The advancement of technology encouraged scientists to think about personalized medicine. In the 1990s, the first gene therapy (Figure 1) was initiated to insert the gene encoding the protein into the cells of the person having hereditary health condition. Collection of patient’s hematopoietic progenitor (or stem) cells, insertion of a healthy gene copy using viral vectors into the collected stem cells, their differentiation and transfer to the patient’s body was performed [6]. After more than 20 years we have an increasing number of approved gene therapy treatments (namely, melanoma therapy [7], lipoprotein lipase deficiency therapy [8], Duchenne muscular dystrophy therapy (FDA release in 2020, https://www.fda.gov/news-events/press-announcements/fda-approves-targeted-treatment-rare-duchenne-muscular-dystrophy-mutation)).

Another direction of biological therapy is the use of **RNA oligonucleotides **(Figure 1). These small RNA molecules are created to hybridize on specific pre-mRNA sites. The hybridization can lead to cleavage and skipping of the exon(s) with pathogenic changes (the use of antisense oligonucleotides) or preserving the exon(s) in mRNA therefore increasing a possibility of producing full-length and functional proteins (the use of splice modulating oligonucleotides) [9]. A perfect example of the latter one is the treatment of spinal muscular atrophy (SMA) which is mainly caused by the deletion of the 7^th^ exon of *SMN1* gene. This gene is modified by *SMN2*, and the main difference between these two genes lies in their DNA sequence: several nucleotide changes in *SMN2* gene determine the predominant synthesis of exon 7-free mRNA transcript. The therapeutic oligoribonucleotides are designed to increase the incorporation of exon 7 in *SMN2* mRNA and therefore partially rescuing the functional SMN protein [10]. This RNA therapy was approved in 2016 (FDA release https://www.fda.gov/news-events/press-announcements/fda-approves-first-drug-spinal-muscular-atrophy).

### Programmable nucleases

From the last decade of the twentieth century cellular processes were further exploited for genome editing. Double strand breaks (DSBs) are naturally occurring events in cells when both DNA strands are cut. However, the DSBs introduction at the specific site is very low therefore to increase the specificity and the efficiency of the DSBs recombination (repair mechanisms of DSBs are discussed later), molecular tools for introduction of the DSBs are required.

**Figure 2.**
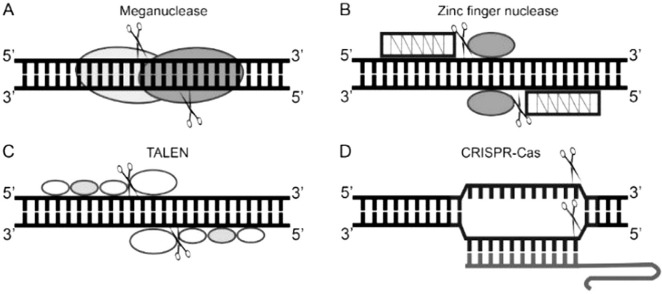
**Schematic representation of programmable nucleases used as genome editing tools.** A – meganuclease consists of two monomers that form a homodimer. B – Zinc finger nuclease consists of *FokI* endonuclease (restriction domain) and a DNA binding module that is formed by varying number of zinc finger motifs. C – TALEN protein also has the restriction domain (*FokI* endonuclease) and a DNA binding module that is formed by a different number of TAL effector protein’s DNA targeting domains. D – CRISPR-Cas9 editing system consists of Cas9 endonuclease and guide RNA molecule that together forms a ribonucleoprotein

Scientists carried out experiments aiming to investigate the characteristics and possible targeting strategies of endonucleases. Early trials with the DNA cutting endonuclease, called **meganuclease** (Figure 2A), showed that this protein not only can precisely recognize a specific, usually more than 14 bp long DNA sequence, but also to cut both of its strands [11, 12]. However, the reprogrammability of the target specificity of meganucleases is time and labor consuming because one specific protein has only one particular target. Nonetheless, meganucleases are being explored and applied in developing treatments for different medical conditions. In 2021, Presicion BioSciences company is using its technology ARCUS^®^ (meganuclease based genome editing) to perform a clinical trial to evaluate the safety and clinical activity of their allogenic CAR T cell approach in treating relapsed or refractory (r/r) Non-Hodgkin Lymphoma (https://investor.precisionbiosciences.com/news-releases/news-release-details/precision-biosciences-receives-notice-allowance-us-patent, also see Table 1).

With more exploration of endonucleases and knowledge of DNA binding domains and gene expression, hybrid nucleases, namely **ZFNs (Zinc Finger Nucleases) **and, subsequently, **TALENs (Transcription Activator-Like Effector Nucleases)**, were designed (Figure 2B-C). These modified endonucleases are similar in structure: they both consist of a DNA binding module (several zinc finger DNA-binding motifs attached together in ZNFs and TAL effector protein’s DNA targeting domains in TALENs) and a cleavage domain of restriction endonuclease *FokI* [13–16]. Although, both nucleases can be modified to introduce DSBs at specific sites by engineering different combinations and number of DNA binding domains (even though the process is time and labor consuming), the off-targets still occur which can increase the cellular toxicity of such endonucleases and the molecular size of engineered protein can complicate their delivery to living cells [17].

The breaking point in creating an affordable and easier to program genome editing tool occurred with **CRISPR-Cas systems**, specifically with CRISPR-Cas9 (DNA endonuclease of type II CRISPRCas systems) experiments (Figure 2D). Studies on the use of this RNA-guided DNA-cutting protein for editing various genomes have been published in 2012–2013. One of the outstanding features of CRISPR-Cas9 system is its genome targeting mechanism: guide RNA (gRNA) is an RNA molecule complex, formed by hybridization of crRNA and tracrRNA, which guides Cas9 endonuclease to a genome target of interest and is rather simply reprogrammable by changing the ribonucleotide sequence without the necessity to modify Cas9 protein [18–20]. In this respect, CRISPR-Cas9 technology became an intensively applied, studied, and engineered genome editing tool which is the state-of-the-art genome-targeting system in medicine, too [21–23]. However, CRISPR-Cas9 system is not ideal. The off-targets are also created by this technology which is one of the shortcomings that can have a negative impact on cellular processes and applicability in developing safe therapeutics for various diseases. The other issue is the requirement of the PAM (protospacer adjacent motifs) sequence to be present in the desired gene target because Cas9 protein cleaves DNA near it which limits the choice of specific DNA target. Aside from these limitations, different CRISPR-Cas systems are being modified to overcome these restrictions and to meet the required features. Nevertheless, genome editing technique provided significant breakthrough in biotechnology, therefore CRISPRCas9 researchers were awarded by the Nobel prize in 2020. Altogether, programmable endonucleases differ in the sequence recognition method, specificity, recognizable sequence properties, simplicity of production, immunogenicity, mode of delivery to the cell (discussed later) [24]. These are one of the defining factors to be considered before applying genome editing tools to investigations and treatment development of different human medical conditions.

## Challenges in developing genome editing strategies for clinical practice

### Enhancing the repair mechanism

**Figure 3.**
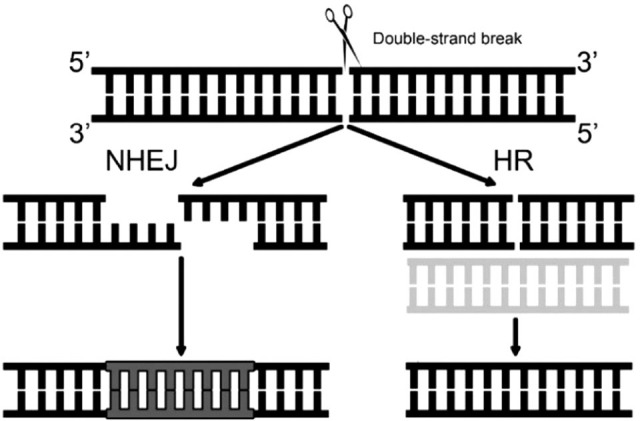
**The main DNA double-strand break repair mechanisms in the cell evoked during genome editing process. **NHEJ – non-homologous end joining is a DNA damage repair mechanism that occurs frequently in the cell and is more error-prone. HR – homologous recombination is a DNA damage repair mechanism that uses DNA template to correct the error which preserves genetic material from undesirable alterations.

The clinical situation is important in choosing genome editing strategy for receiving expected results – activation or inactivation of the gene. By creating DSB in the genome area of interest with programmable nuclease, one of the cell’s genome repair mechanisms are engaged. When the goal is to inactivate gene, error-prone NHEJ mechanism (Figure 3) is expected. It usually disrupts a specific genome sequence because NHEJ corrects DSBs without using a DNA template resulting in insertions and deletions. Different situation arises when the goal is to correct existing change in the DNA sequence by HR mechanism (Figure 3). Studies have shown that HR damage repair is rare comparing to predominant NHEJ [25,26], therefore the need to increase the efficiency of HR is significant. A donor DNA molecule with correct nucleotide sequence is one of the integral elements in homology-directed repair mechanism where it is used as a template by cell’s HR proteins to restore the damage [25,26]. Therefore, the donor DNA itself and the features of it are important. According to the literature, single-stranded donor DNA oligonucleotides as well as linearized plasmid templates can influence HR efficiency, and the longer homology arms at 5’ and 3’ ends of donor DNA can enhance the HR [27–29]. Additionally, promoting the expression of main HR proteins is a known method to increase frequency of homology-directed repair [30]. However, the latter approach is questionable in the sense of unwanted alterations of gene expression when genome editing is studied for clinical applications. Recently a prime-editing technology was described by Anzalone and his colleagues (2019) where different types of genome changes can be introduced by prime editor (PE; it uses a prime editing RNA as a guide and the protein itself consists of reverse transcriptase fused with RNA-programmable nickase which is a part of specific Cas9 protein) without double stranded breaks or even donor DNA [31]. This new technology is a promising tool for developing genome editing therapies for various genetic diseases.

### Delivering to living cells

**Figure 4.**
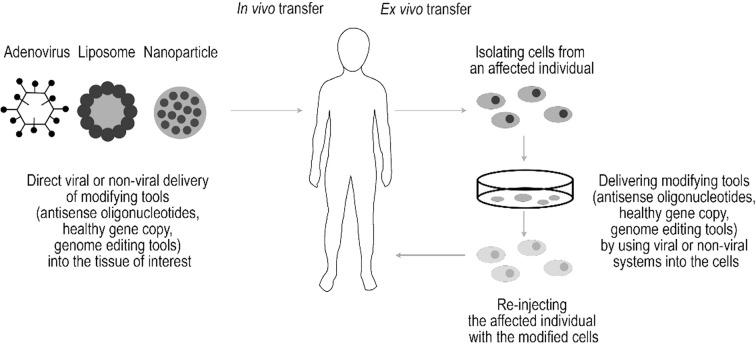
***In vivo***** and *****ex vivo***** transfer. **In vivo transfer is based on direct delivery of antisense therapy, gene therapy or gene editing tools in the tissue of interest using viral or nonviral delivery system. During ex vivo transfer, cells from the affected individual are isolated, modified using the specific technology, and only then reinjected in the affected individual.

The transfer of the genome editing systems to cells is a considerable and tricky part of the experiment design. For clinical application, the process may be performed *ex vivo *in cell culture before transplanting cells back into the body or *in vivo* when therapeutic cargoes are delivered directly into the body. When **transferring *****ex vivo*** (Figure 4), it is important for cells to survive genetic manipulations in culture and then resettle when they are returned. *Ex vivo* experiments are often performed with the haematopoietic system (common stem cells) due to relatively easy access of the cells, the high clinical experience with their cultivation and various manipulations [32,33]. When manipulating cultured cells, the main barrier for the genome editing cargo (in case of the CRISPR-Cas9 based genome manipulation, the delivery mode could consist of Cas9 protein’s DNA/mRNA and gRNA or a full ribonucleoprotein and gRNA) is cell membrane which can be passed in nonviral or viral way: electroporation, microinjection, lipofection, various viral vectors, nanoparticles, etc. [34]. Depending on the delivery system, genome editing efficiency differs with viral systems being usually more effective [34,35].

During ***in vivo *****transfer **(Figure 4), the programmable nuclease payload is transmitted through the body into the cells. The issue here is that the therapeutic elements must reach the target and still be stable and functional after passing different environments. Therefore, various viral and nonviral delivery systems are being studied and developed to reach the wanted effect. Commonly used viral systems are adenoviral (AV), adeno-associated viral (AAV), also lentiviral vectors [36]. The main concern for using viral systems is the immune response in human body. Viral vectors that integrate the DNA sequence into the genome are more dangerous than those carrying the nuclease [36]. When working with viral vectors, all work safety and precautionary requirements must be observed. AAVs that integrate into a certain “safe” area of the genome have become mainly used vectors. Over 10 types of AAV have been identified that have different affinities for organs [37].

Immunogenicity is one of the reasons why nonviral methods are extensively created and improved. Lipid and gold nanoparticles as well as direct modification of gRNA and Cas protein by conjugating them with cell-penetrating peptides are several examples of tissue cells without the use of viral systems [34,36]. Although virus-free and synthetic delivery systems reduce the risk of stimulation of the immune reaction, the possibility of adaptive immune response remains, and one of several other hurdles is that transfection of target’s cells is relatively low compared to viral systems [36,38]. All in all, *ex vivo* and *in vivo* therapy with diverse delivery systems, different genome editing modes face various obstacles. For this reason, designing the genetic manipulation strategy for therapeutic purposes is a difficult and complex process. Scientific efforts are involved in this process, and new discoveries emerge in it continually. 

### Examples of side effects of genome editing

**The viability of cells after genome editing** could depend on the effect of the modified gene on the cell. If the edited gene positively affects cell proliferation (e.g., an *IL2RG* gene whose pathogenic variants result in severe immunodeficiency), then the cells with the edited genome will dominate other cells and will have a therapeutic effect [39]. If the edited gene does not have such an effect, there will be no dominance, the effect of “edited” cells on the symptoms of the disease will be poor (e.g., chronic granulomatous disease due to pathogenic variants of the phagocyte oxidase proteins’ genes) [40]. On the other hand, there are diseases, whose clinical symptoms could be eased by 1% of functioning cells (e.g., haemophilia B) [41].

**The stability of corrected genome** is one of the issues caused by genome editing off-target cleavage since the cell’s genome will be changed irreversibly and any errors will result in long-term effects [42,43]. Nonspecific cutting sites, which can be influenced by cell type, DNA methylation, overall genetic manipulation design, and disturbed process of cell’s natural DSB repairing mechanism can increase the risk of unbalanced cellular processes [42–44]. In this regard, editing of the genome in target areas with low risk of formation of DSBs in nonspecific genome sites does not appear to be very dangerous, but the risk of a partial donor DNA integration in the genome causing various allele changes may have unexpected consequences [45]. The risk of the formation of breaks in nonspecific locations is reduced by *in silico *analysis of the genome and calculating off-targets, choosing a maximum specific area during the development of the genome editing strategy [46]. To reduce genomic editing events in nonspecific areas, even more specific genetic bioengineering tools are being developed, which could be able to correct single-nucleotide changes without additional separate parts being introduced together into the cell or without creating DSBs (such as prime editors mentioned before).

### Bioethical issues

The greatest concern related to significant advances in genome editing technology is the consequences of editing a human embryo. In 2015 a moratorium on such experiments was proposed, but the groups of scientists published the results of various studies on human embryos one after another, despite controversial assessments by the scientific society. Scientific arguments about the benefits of such research are faced with an objective lack of fundamental knowledge, anticipating potential consequences, lack of legal regulation and subjective fears about human selection, the emergence of “invasive mutants” and the creation of bio-weapons [47].

In 2017 The American Society for Human Genetics (ASHG) has published an expert opinion on the issue of editing the human embryonic genome [48]. It stated that at this time, given the nature and number of unanswered scientific, ethical, and policy questions, it is inappropriate to perform germline gene editing that culminates in human pregnancy. Also, their experts’ opinion on *in vitro* germline genome editing is that there is no reason to prohibit this editing on human embryos and gametes, with appropriate oversight and consent from donors, to facilitate research on the possible future clinical applications of gene editing, and there should be no prohibition on making public funds available to support this research. Moreover, according to the statement future clinical application of human germline genome editing should not proceed unless, at a minimum, there is (a) a compelling medical rationale, (b) an evidence base that supports its clinical use, (c) an ethical justification, and (d) a transparent public process to solicit and incorporate stakeholder input. 

When the report on the birth of twin sisters with edited genomes in China (2018) reached the authorities, it was reaffirmed that ASHG holds the position statement where *in vitro* human germline genome editing is allowed while genome editing that involves human pregnancy is considered as misdemeanor (press release at ASHG website). This event confirmed that genome editing in humans for clinical purposes is not ready and faces various legal and bioethical issues and gaps.

## Genome editing in clinical practice

### Immune system and malignant tumors

**Infectious disorders.** Intensive research is ongoing in many areas of medicine and one of them is infectious diseases. Genome editing could potentially be useful for treating viral diseases by removing the sequence of viral genome integrated in hosts’ cell’s genome or by modifying the hosts’ cellular receptor necessary for the virus to infect the target cells. These strategies using *ex vivo* or *in vivo* approach (discussed earlier) were tested in experiments with human immunodeficiency virus (HIV) [49,50] (Table 1). The strategy of inactivating the *CCR5* gene (encoding chemokine receptor 5) in cells, thus preventing the HIV virus from integrating into the cell and destroying it, was suitably applied [50,51]. Recently the *CCR5* knock-out approach received an immediate attention after it was unethically and illegally practiced in genomes of two human embryos using CRISPR-Cas9 technology (human embryo treatment approach) and later twin sisters were born [52].

**Malignant tumors.** Genome editing is also being extensively investigated for treating malignant tumors (Table 1). An example of a successful experiment could be the CAR-T cell (chimeric antigen receptor T lymphocyte cell) therapy. This system seeks to develop T lymphocytes able to efficiently recognize and fight cancer cells. The developing process begins with T lymphocytes of a patient suffering with cancer being transferred with chimeric protein receptor genes expressed by malignant cells, thus ensuring their recognition and destruction by immune cells [53]. The T cells could also be passed through several other changes: to avoid graft-versus-host reaction, T cell genome is edited by inactivating the genes coding T cell receptor (TCR), T cells could also be altered to eliminate HLA-I antigens thereby reducing immunogenicity, as well as disruption of CD52 protein gene could increase T cell resistance to chemotherapeutic agent alemtuzumab [54,55]. Alterations of endogenous TCR and HLA-I elimination create a possibility to develop universal (not patient-specific) CAR T cells for the treatment of various types of tumors [55].

### Approaching hereditary diseases

In the case of hereditary diseases (Table 1), pathogenic gene changes may result in the acquisition or loss of function of coded protein. Depending on the nature of the disorder, the principles of genome editing vary. 

**Autosomal dominant disorders.** When point pathogenic variant leads to gain of a harmful function, as in the case of achondroplasia inherited in autosomal dominant manner, it would suffice to form a double-stranded break of a mutated gene allele, which would create an insertion or deletion after nonhomologous end joining process (NHEJ) leading to frameshift and truncated protein that do not affect the person’s phenotype. This type of pathogenic variant could also be corrected by inducing homologous recombination to restore the wild type phenotype (for example achondroplasia [56]). Hereditary diseases whose pathogenesis involves prolongation of short tandem repeats (STR), a two-site cutting on both sides of the elongated sequence could be used to remove it from the gene allele. Also, when the STR creates harmful protein which disrupts normal functions and it could benefit from elimination of mutant protein, the NHEJ inducing strategy could be considered as it was investigated for Huntington’s disease [57].

**Autosomal and X-linked recessive disorders.** A more complicated situation is with recessive diseases when the protein function is lost because both alleles possess pathogenic changes. The non-homologous end joining, being more frequently exploited in cells, would not be effective as it would lead to a loss of protein function. Therefore, together with the programmable endonuclease system, a donor DNA fragment, which is necessary for homologous recombination, with unmodified gene sequence is one of the elements to be introduced into the cell and used by proteins performing HR process for repairing the pathogenic variants [30]. Moreover, there are recessive diseases that could benefit from the destruction or excision of the exon(s) with premature endogenous codon, thus restoring most of the protein sequence and at least in part the function as was shown by the studies performed on cells derived from Duchenne muscular dystrophy patient [58]. Although complicated, correction of chromosomal changes is also considered and investigated as a target for genome editing technologies [59]. Research conducted in recent years demonstrates the potential of genome editing in the prevention and treatment of complex diseases, too (for example, Alzheimer’s disease [60]). All in all, the experiments exploring different diseases in cells or animal models throughout the years yielded hopeful results for the genome editing tools directed to treatment of various human pathologies, including severe combined immunodeficiency (SCID) [61], different ophthalmology related conditions [62], cystic fibrosis [63], and many others.

**Table 1.** **Biological therapy medicines approved or in an approval process in the European Union and medicines at a preclinical state in the European Union and the United States of America. **The medicines in this table depict a part of the biological therapy treatments that are approved or in preclinical state. More information about these treatments and their state could be found in https://crisprmedicinenews.com/, https://www.ema.europa.eu/en, https://clinicaltrials.gov/ct2/home.

Approved or in approval process (in European Union)					
Disease	Treatment target	Therapy type	Medicine name	State of the medicine	Source of information about the medicine
**Hereditary diseases**					
Metachromatic leukodystrophy	*ARSA* gene	Gene therapy	Libmeldy	Authorised (approved)	https://www.ema.europa.eu/en/medicines/human/EPAR/libmeldy
Severe combined immunodeficiency due to ADA deficiency	*ADA* gene	Gene therapy	Strimvelis	Authorised (approved)	https://www.ema.europa.eu/en/medicines/human/EPAR/strimvelis
Inherited retinal dystrophy (retinitis pigmentosa)	*RPE65* gene	Gene therapy	Luxturna	Additional monitoring	https://www.ema.europa.eu/en/medicines/human/EPAR/luxturna
Hereditary transthyretin amyloidosis	*TTR* gene	Antisense therapy	Tegsedi	Authorised (approved)	https://www.ema.europa.eu/en/medicines/human/EPAR/tegsedi
Acute hepatic porphyria	*ALAD* gene	Antisense therapy	Givlaari	Authorised (approved)	https://www.ema.europa.eu/en/medicines/human/EPAR/givlaari
Spinal muscular atrophy (type 1, 2 and 3)	*SMN2* gene	Antisense therapy	Evrysdi	Authorised (approved)	https://www.ema.europa.eu/en/medicines/human/EPAR/evrysdi
Spinal muscular atrophy (type 1)	*SMN1* gene	Gene therapy	Zolgensma	Conditional approval	https://www.ema.europa.eu/en/medicines/human/EPAR/zolgensma
Beta thalassaemia	*HBB* gene	Gene therapy	Zynteglo	Under evaluation by EMA	https://www.ema.europa.eu/en/medicines/human/referrals/szynteglo
Early cerebral adrenoleukodystrophy	*ABCD1* gene	Gene therapy	Skysona	Recommendation for EMA to grant a marketing authorisation	https://www.ema.europa.eu/en/medicines/human/summaries-opinion/skysona
**Malignancies**					
Diffuse large B-cell lymphoma, primary mediastinal large B-cell lymphoma	Gene encoding CAR protein	Gene therapy	Yescarta	Authorised (approved)	https://www.ema.europa.eu/en/medicines/human/EPAR/yescarta
B-cell acute lymphoblastic leukaemia, diffuse large B-cell lymphoma	Gene encoding CAR protein	Gene therapy	Kymriah	Authorised (approved)	https://www.ema.europa.eu/en/medicines/human/EPAR/kymriah
**Preclinical state (in European Union, United States of America)**					
**Disease**	**Treatment target**	**Therapy type**	**Medicine name**	**State of the medicine**	**Source of information about the medicine**
**Hereditary diseases**					
Mucopolysaccharidosis (type 1)	*IDUA* gene	Gene editing (Zinc finger nuclease)	SB-318	Active clinical trial, not recruiting potential participants yet	https://clinicaltrials.gov/ct2/show/NCT02702115?term=NCT027021 15&draw=2&rank=1 https://crisprmedicinenews.com/clinical-trial/mucopolysaccharidosis-type-i-mps-i-nct02702115/
Mucopolysaccharidosis (type 2)	*IDS* gene	Gene editing (Zinc finger nuclease)	SB-913	Active clinical trial, not recruiting potential participants yet	https://clinicaltrials.gov/ct2/show/NCT03041324?term=gene+editing&recrs=d&draw=2&rank=3 https://crisprmedicinenews.com/clinical-trial/mucopolysaccharidosis-ii-mps-ii-nct03041324/
Transfusion dependent Beta-Thalassemia, Sickle Cell Disease	*BCL11A *gene	Geneediting (CRISPR-Cas	CTX001	Recruiting participants	https://clinicaltrials.gov/ct2/show/NCT03655678?term=CTX001&draw=2&rank=3 https://clinicaltrials.gov/ct2/show/NCT03745287?term=CTX001&dr aw=2&rank=2
Leber Congenital Amaurosis (Type 10)	*CEP290* gene	Gene editing (CRISPR-Cas9)	EDIT-101	Recruiting participants	https://clinicaltrials.gov/ct2/show/NCT03872479?term=EDIT-101&draw=2&rank=1 https://crisprmedicinenews.com/clinical-trial/leber-congenital-amaurosis-nct03872479/
Hereditary Transthyretin Amyloidosis	*TTR* gene	Gene editing (CRISPR-Cas9)	NTLA-2001	Recruiting participants	https://clinicaltrials.gov/ct2/show/NCT04601051?term=NCT046010 51&draw=2&rank=1 https://crisprmedicinenews.com/clinical-trial/transthyretin-amyloidosis-attr-nct04601051/
**Infectious diseases**					
Refractory herpetic viral keratitis	Herpes simplex virus type I genome	Gene editing (CRISPR-Cas9)	BD111	Active clinical trial, not recruiting potential participants yet	https://clinicaltrials.gov/ct2/show/NCT04560790?term=gene+editing &draw=2&rank=1 https://crisprmedicinenews.com/clinical-trial/herpes-simplex-virus-refractory-keratitis-nct04560790/
Human Immunodeficiency Virus Infection	*CCR5* gene	Gene editing (CRISPR-Cas9)	CCR5 gene modification	Unknown (A study on whose the status has not been last verified within the past 2 years)	https://clinicaltrials.gov/ct2/show/NCT03164135?term=NCT03164135&draw=2&rank=1 https://crisprmedicinenews.com/clinical-trial/human-immunodeficiency-virus-infection-hiv-nct03164135/
**Malignancies**					
Relapsed or refractory renal cell carcinoma	*TRAC* locus	Gene editing (CRISPR-Cas9)	CTX130	Recruiting participants	https://clinicaltrials.gov/ct2/show/NCT04438083?term=gene+editing&recrs=a&draw=2&rank=9 https://crisprmedicinenews.com/ clinical-trial/renal-cell-carcinoma-rcc-nct04438083/
Gastro-Intestinal Cancer	*CISH* gene	Gene editing (CRIPSR-Cas9)	TumorInfiltrating Lymphocytes (TIL)	Recruiting participants	https://clinicaltrials.gov/ct2/show/NCT04426669?term=gene+editing &draw=2&rank=8 https://crisprmedicinenews.com/clinical-trial/gastro-intestinal-cancer-gi-nct04426669/
Human Papillomavirus- Related Malignant Neoplasm	Human papillomavirus genes encoding proteins E6 and E7	Gene editing (TALENs)	T27 and T512	Recruiting participants	https://clinicaltrials.gov/ct2/show/NCT03226470?term=NCT03226470&draw=2&rank=1 https://crisprmedicinenews.com/clinical-trial/human-papillomavirus-hpv-related-cervical-cancer-nct03226470/
Non-Hodgkin Lymphoma	*TRAC* locus	Gene editing (meganuclease)	PBCAR19B	Recruiting participants	https://clinicaltrials.gov/ct2/show/study/NCT04649112 https://investor.precisionbiosciences.com/news-releases/news-release-details/precision-biosciences-receives-notice-allowance-us-patent https://crisprmedicinenews.com/clinical-trial/haematologic-malignancy-non-hodgkin-lymphoma-nhl-nct04649112/
Metastatic Non-small Cell Lung Cancer	*PDCD1* gene	Gene editing (CRISPR-Cas9)	PD-1 Knockout T Cells	Completed	https://clinicaltrials.gov/ct2/show/NCT02793856?term=NCT027938 56&draw=2&rank=1 https://crisprmedicinenews.com/clinical-trial/metastatic-non-small-cell-lung-cancer-nsclc-nct02793856/

## Conclusion

The potential of genome editing technologies in medicine is tremendous. Experiments are and will be helping to analyze early embryogenesis, develop cellular models of various diseases, analyze drug efficacy and toxicity, and develop devices of “precise medicine”. Innovative ways of treating patients with various conditions and the approved new therapeutic applications show promising results every year. Overall genome editing tools provide hope for their future adjustment in medicine when technology will be improved, and bioethics issues will be addressed.
